# Long-term economic and welfare consequences of Ménière’s disease: a Danish nationwide matched cohort study, 2002–2016

**DOI:** 10.1007/s00405-026-10140-z

**Published:** 2026-05-05

**Authors:** Casper Grønlund, Sören Möller, Helle Collatz Christensen, Mikkel Porsborg Andersen, Christian Torp-Pedersen, Louise Devantier, Bjarki Ditlev Djurhuus

**Affiliations:** 1 Department of Ear, Nose, Throat, and Maxillofacial Surgery, Zealand University Hospital, Køge, Denmark; 2 Department of Public Health, University of Southern Denmark, Odense, Denmark; 3 The Prehospital Center, Region Zealand, Næstved, Denmark; 4 Department of Health Science and Technology, Aalborg University, Aalborg, Denmark; 5 Steno Diabetes Center Copenhagen, Copenhagen University Hospital, Copenhagen, Herlev, Denmark; 6 Department of Public Health, University of Copenhagen, Copenhagen, Denmark; 7 Department of Ear, Nose, Throat, Aarhus University Hospital, Aarhus, Denmark; 8 Faculty of Health and Medical Sciences, University of Copenhagen, Copenhagen, Denmark

**Keywords:** Ménière’s disease, Vertigo, Income, Cost, Employment status

## Abstract

**Purpose:**

To assess the long-term economic and welfare burden of Ménière’s disease, focusing on healthcare costs, income, and reliance on social benefits before and after the first hospital-recorded diagnosis.

**Methods:**

We conducted a nationwide matched cohort study in Denmark, involving 5,434 patients with a hospital-recorded Ménière’s disease diagnosis later confirmed by an otorhinolaryngologist and 16,302 matched controls based on age, sex, civil status, municipality, and index year defined by the first hospital registration of Ménière’s disease. The mean age was 59 years, and 56% were females. The study assessed outcomes such as annual healthcare costs, income disparities, and social benefit receipt from ten years prior to ten years after diagnosis.

**Results:**

Annual healthcare costs were higher in patients with Ménière’s disease after diagnosis, peaking at index year (EUR 3,818 vs. EUR 1,862 in controls). Patients with Ménière’s disease also showed a sustained decline in annual income, with the largest decline at year 8 after diagnosis (EUR − 1.908 compared to controls). At index year, 11% of patients transitioned to sickness benefits (vs. 3% of controls). From index year to 120 months post-diagnosis, disability pension rates increased from 10% to 18% in patients with Ménière’s disease, whereas they remained almost stable going from 10% to 9% among controls. Similarly, participation in the flexible job scheme rose from 4% to 10% in Ménière’s disease compared with 2% to 4% in controls.

**Conclusion:**

Ménière’s disease was associated with persistently higher healthcare costs, reduced income, and greater reliance on social benefits compared with matched controls, particularly evident around and after the time of diagnosis. These findings highlight the substantial long-term economic and welfare burden of the disease.

**Supplementary Information:**

The online version contains supplementary material available at 10.1007/s00405-026-10140-z.

## Introduction

Ménière’s disease is a chronic inner ear disorder characterised by sudden spontaneous episodes of vertigo, unilateral hearing loss, tinnitus or aural fullness. Over a range of years, the hearing loss usually progresses to complete or near-complete deafness [1]. The incidence ranges from 8.2 to 157 per 100,000 individuals per year, and the prevalence from 3.5 to 513 per 100,000 individuals depending on geographic location [2–7]. The disease usually develops in the fourth or fifth decade of life and can cause disabling symptoms that adversely affect daily functioning, employment, and overall quality of life [1]. Its unpredictable progression and the absence of a cure often result in repeated healthcare visits and prolonged monitoring. Although the clinical presentation and underlying mechanisms of Ménière’s disease have been extensively investigated [1, 8–13], less is known about its wider societal effects, including healthcare utilisation patterns, income loss, and reliance on social support systems.

Understanding the economic and welfare burden of Ménière’s disease is essential for healthcare planning, patient counselling, and policy making. However, only a few population-based studies have comprehensively examined long-term trajectories of healthcare costs, employment outcomes, and public support in individuals diagnosed with Ménière’s disease compared to the general population [14–17].

The aim of this nationwide register-based study, utilising comprehensive registry data from Denmark, was to measure the economic and welfare impacts of Ménière’s disease. We examined its effects across three areas: annual healthcare costs in primary and secondary care, annual personal income, and the distribution and progression of dependence on social benefits.

## Methods

### Ethics

In Denmark, register-based studies conducted for scientific research and statistics purposes with societal importance do not require ethical approval or informed consent [18]. However, the study was approved by the data responsible institute, the Capital Region of Denmark (approval number: P-2019-264), in accordance with the Danish Data Protection Act and the General Data Protection Regulation (GDPR).

### Data sources

This nationwide register-based cohort study uses data from multiple Danish National Health and Social Registers [19]. The following registers were included:

- The National Patient Register, for hospital contacts, diagnoses, and associated costs of hospital outpatient visits and hospital admissions [19].
- The National Health-Service Register, for reimbursement data from office-based specialist care [20].
- The Civil Registration system, for demographic information [21].
- The Income Registers, for income-data [22].
- The Register of Causes of Death, used to track mortality [23].
- The Danish Register for Evaluation of Marginalization (DREAM) database to monitor social benefit receipt over time [24].
- The National Population Education Register [25].

For every participant, a unique anonymized personal identification number was used to merge data from different datasets.

### Study population and matching

Patients with Ménière’s disease (ICD-10 code H810) diagnosed by an otorhinolaryngologist between 2002 and 2016 were identified in the Danish National Patient Register. The period from 2002 to 2016 was chosen because data on prices were available only for those specific years. The first occurrence of the diagnosis, regardless of the diagnosing specialty, was considered the time of diagnosis. The index year thus represents the first hospital-based registration of H810 rather than true disease onset. However, later confirmation by an otorhinolaryngologist was required for inclusion in the study. To ensure the inclusion of incident cases, a wash-out period from 1995 to 2002 was applied. Patients with a registration during this period were excluded, ensuring that primarily only newly diagnosed cases from 2002 onward were included. Since the Danish National Health Service Register, which records office-based specialist visits, does not report diagnostic codes to national registers and is therefore unavailable, patients treated solely in private practice without hospital confirmation were not eligible for inclusion.

For each patient, three control individuals were matched based on gender, age, index year, civil status, and municipality code. The control individuals were randomly selected from the Danish Civil Registration system during the study period. It was ensured that the control group had no recorded diagnoses of either Ménière’s disease (H810), benign paroxysmal positional vertigo (H811) or vestibular neuritis (H812) in the National Patient Register. Exact matching was used for gender, municipality, and civil status, while exposure density matching was applied for age (within a one-year caliper) and index year (within a two-year caliper).

For each individual, we determined whether they had one or more of the major chronic disease groups using ICD10 codes registered in the National Patient Register: chronic obstructive pulmonary disease, cerebrovascular disease, heart failure, any malignancy, liver disease, and diabetes mellitus with or without complications. This allowed adjustment for comorbidities in subsequent analyses. Additionally, we identified each individual’s highest level of education at the index year to enable adjustment in later analyses.

### Healthcare costs, income, and social benefits

Annual average healthcare costs per individual were estimated using diagnosis-related group (DRG) tariffs from the Danish National Patient Register [26]. Costs associated with office-based specialist care were obtained from gross fees listed in the Danish National Health Service Register [27].

Annual mean gross personal income was obtained from the National Income Register (variable PERSONINDK). Gross personal income included all taxable earnings, such as wages, pension payments, disability benefits, and other public transfers. All income figures were adjusted for inflation relative to 2015 using the Danish CPI and converted into EUR.

From the DREAM database [24], weekly records of public transfer payments were combined into a monthly overview. For each month, the most common support type (the category with the most days) was assigned; individuals without registered support were categorised as “No public support”.

### Statistical analysis

Baseline characteristics were summarised with categorical variables shown as counts and proportions, and continuous variables as medians with interquartile ranges. A p-value < 0.05 was considered statistically significant, and results were reported with 95% confidence intervals (95%-CI).

All regression analyses were performed using single longitudinal models including time since diagnosis as a covariate, rather than estimating separate models for each year. Repeated annual observations contributed by the same individual were accounted for using cluster-robust standard errors.

Annual healthcare costs and income were calculated separately for each group and year. Foregone earnings were defined as the difference in annual income between the Ménière’s and control groups. Total costs were calculated as the sum of healthcare costs and foregone earnings, with differences between groups obtained by subtracting the control group’s costs from those of the Ménière’s group. Formal hypothesis testing and confidence intervals were not applied because the analyses focused on long-term patterns and absolute differences in total costs. All costs are reported as mean annual values in euros (EUR).

Adjusted healthcare costs were estimated through a two-step process. First, the likelihood of incurring any healthcare expenditure in a given year was modelled using a generalised linear model with a logistic link. Second, among individuals with non-zero costs, differences in cost levels over time between cases and controls were estimated using a generalised linear model with a gamma distribution and a log link.

Adjusted income was analysed using a linear regression model that included an interaction between case status and time since diagnosis. These analyses, along with the examination of social benefits, were limited to individuals aged 18–64 years to reflect the population eligible for employment and social support schemes.

Group differences in the monthly distribution of support types from 10 years before to 10 years after diagnosis were visualised using proportional stacked bar plots. To formally assess these differences, a multinomial logistic regression was applied to estimate the association between Ménière’s disease and subsequent labour market outcomes 8 years after the index year. The reference category was ‘No public support’.

All models were adjusted for age, sex, municipality, civil status, education level, and chronic diseases (as defined by Charlson comorbidity index scores (CCI)). Although a matched design was used, we did not apply conditional logistic regression, as this method does not permit adjustment for repeated measurements. Instead, we employed (non-conditional) regression models to account for repeated measurements over time. We also adjusted for the matching variables to prevent bias, since not doing so could attribute differences to the exposure rather than the matching itself. For each calendar year, individuals contributed proportionally to the fraction of the year they were alive. For example, a person who died halfway through a year contributed 0.5 person-years. We did not extrapolate income or costs beyond the date of death. Instead, averages reflect actual experience per person-year alive. Extreme values were addressed using winsorisation at the 97.5th percentile. All monetary values were inflation-adjusted to August 2015 using the consumer price index (CPI) and converted to EUR at that time (1 EUR = 7.46 DKK).

Because the comorbidity burden was generally low (90% of both patients and controls had a CCI score of 1 or lower), we did not match for comorbidities. Instead, we conducted a sensitivity analysis limited to individuals with a CCI score of 0 (comprising 74% of patients and 75% of controls). For this subgroup, overall patterns remained consistent with the main analysis, confirming that the findings were not influenced by differences in comorbidity. As expected, absolute levels of healthcare expenditures and social benefit use were slightly lower in this subgroup, but the reduction was similar for both patients and controls.

All statistical analyses were performed using R (version 4.3.3) [28].

## Results

In total, 5,434 patients with Ménière’s disease were identified. Please refer to supplementary 2, “Flowchart of patient enrolment”, for details of the inclusion process. A matched control group of 16,302 patients was also identified. The mean age was 59 years, and 56% were female (please see Baseline Characteristics in Table 1).

**Table 1:** Baseline characteristics of patients with Ménière’s disease and matched controls in Denmark from 2002 to 2016

**Characteristic**	**Control** N = 16,302^*1*^	**Meniere’s disease** N = 5,434^*1*^
Gender		
Male	7,221 (44%)	2,407 (44%)
Female	9,081 (56%)	3,027 (56%)
Age	59 (48, 69)	59 (48, 69)
Region of Denmark		
Capital Region of Denmark	5,067 (31%)	1,689 (31%)
Central Jutland	2,607 (16%)	869 (16%)
Northern Jutland	1,398 (8.6%)	466 (8.6%)
Region Zealand	2,856 (18%)	952 (18%)
Region South	4,374 (27%)	1,458 (27%)
Civil status		
Marriage or registered partnership	10,608 (65%)	3,536 (65%)
Alone	5,694 (35%)	1,898 (35%)
Education		
1 Primary	45 (0.3%)	18 (0.3%)
2 Lower secondary	5,129 (33%)	1,691 (32%)
3 Upper secondary	6,418 (41%)	2,198 (42%)
5 Short cycle tertiary	665 (4.2%)	197 (3.7%)
6 Bachelor or equivalent	2,399 (15%)	823 (16%)
7 Master or equivalent	996 (6.3%)	297 (5.7%)
8 Doctoral or equivalent	54 (0.3%)	26 (0.5%)
9 Not elsewhere classified	17 (0.1%)	6 (0.1%)

^*1*^n (%); Median (Q1, Q3)

### Raw healthcare costs by sector

**Fig. 1:**
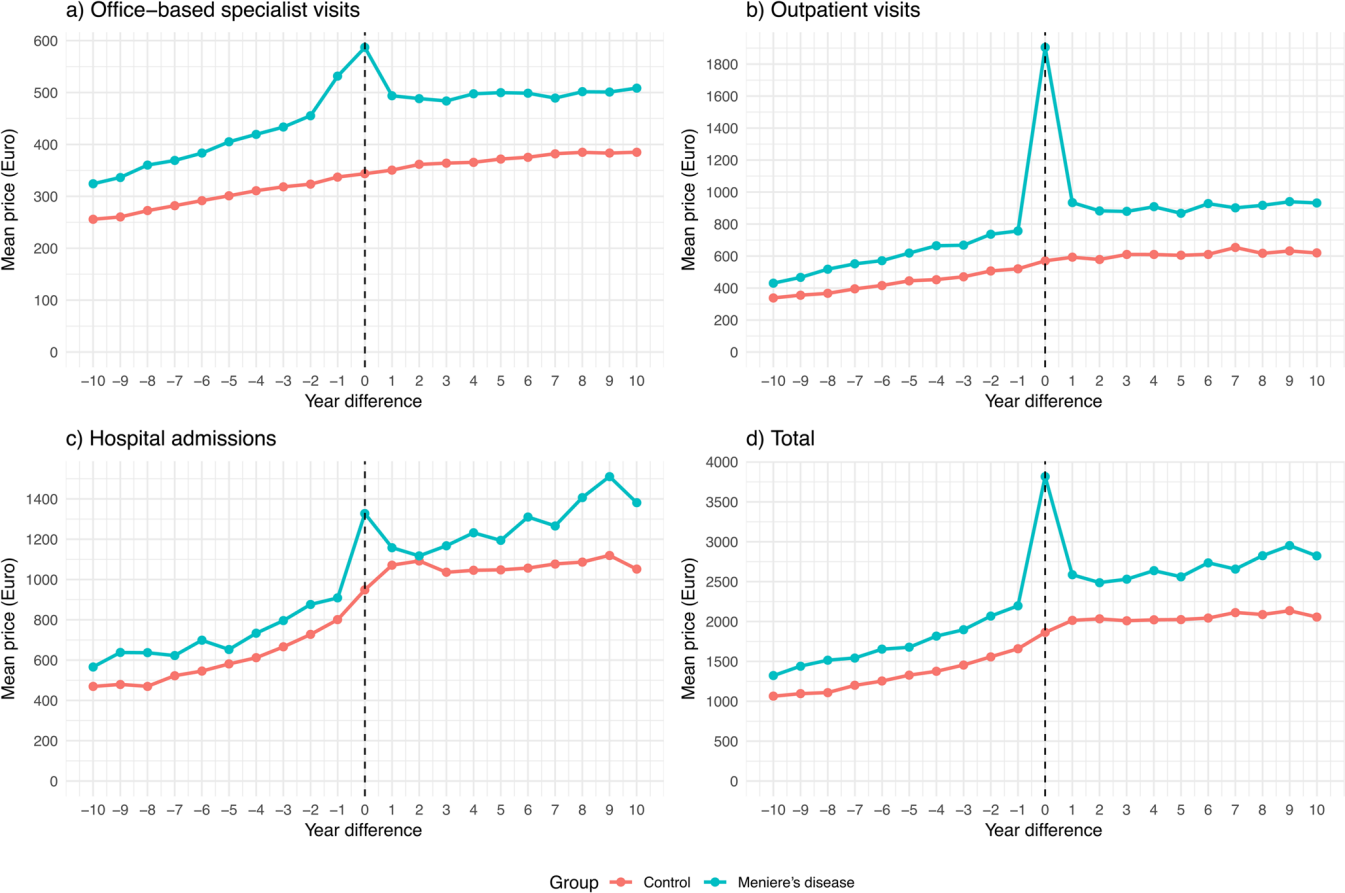
Annual healthcare costs of patients with Ménière’s disease compared to controls in Denmark 2002–2016: office-based specialist visits (**a**), hospital outpatient visits (**b**), hospital admissions (**c**), and total (**d**)

Figure 1 shows the raw data for average annual health costs in EUR, divided into office-based specialist visits, hospital outpatient visits, and hospital admissions for patients with Ménière’s disease compared to controls.

Among individuals with Ménière’s disease, costs related to office-based specialist visits increased in the years preceding diagnosis. After diagnosis, costs declined but remained consistently higher than those of the control group throughout the entire follow-up period. Patients with Ménière’s disease also experienced a significant rise in hospital outpatient-visit costs in the year of diagnosis, peaking at EUR 1,907. Following diagnosis, hospital outpatient-visit costs decreased substantially but stayed persistently elevated compared to the control group throughout the follow-up period. Concerning hospital admission costs, both groups showed a gradual increase leading up to the index year. However, among patients with Ménière’s disease, costs rose more sharply and peaked in the index year, reaching EUR 1,332. Although a slight decline was observed in year one, hospital admission costs for the Ménière’s group remained consistently higher than those of the control group throughout the entire follow-up period, with a continued upward trend up to year 10.

Additional stratified plots by age and gender on healthcare costs are included in the supplementary materials.

### Total economic burden: Healthcare costs and foregone earnings

Supplementary 1 shows total healthcare expenditures for patients with Ménière’s disease compared with the matched control group. Patients with Ménière’s disease experienced a consistent annual increase in healthcare costs and lost earnings after diagnosis compared to controls, with costs reaching a peak of EUR 2,316 in year 8.

For the subpopulation with a CCI of 0, a similar pattern appeared. Once again, patients with Ménière’s disease peaked at year 8 post-diagnosis at EUR 2,355.

### Adjusted healthcare expenditures across hospital admissions, hospital outpatient visits, and office-based care settings

**Fig. 2:**
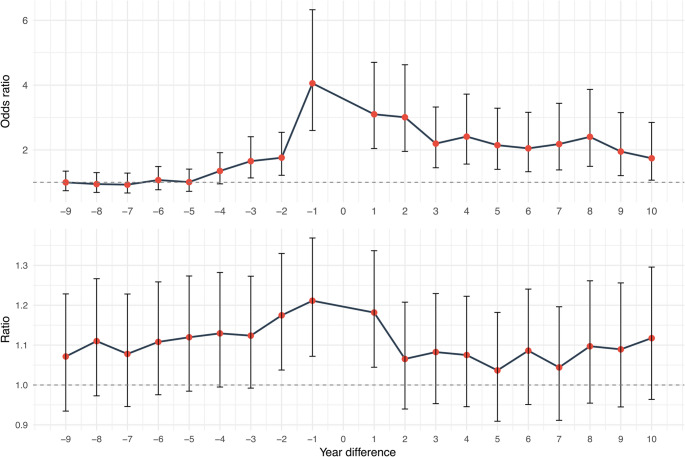
The graphs show (**a**) the odds ratios for having any healthcare expenditure the given year in patients with Ménière’s disease vs. controls, Denmark 2002–2016, and (**b**) the relative cost ratios for total healthcare expenditures the given year (if expenditure in the given year > 0) in patients with Ménière’s disease compared with controls, by year relative to diagnosis, Denmark 2002–2016

The upper part of Fig. 2 shows the likelihood of undergoing a healthcare visit, while the lower part depicts the actual cost ratio among those with visits after adjustments. Figure 2 indicates that individuals with Ménière’s disease had significantly more healthcare visits from one year before to ten years after diagnosis compared to controls, peaking at one year prior to diagnosis (OR = 4.05, 95%-CI: 2.59–6.32). Additionally, for those who incurred costs in the groups, the cost ratio was higher in the Ménière’s disease group from two years prior (ratio: 1.17, 95%-CI 1.04–1.33), one year earlier (ratio: 1.21, 95%-CI 1.07–1.37), and one year after (ratio: 1.18, 95%-CI 1.04–1.36).

### Impact of Ménière’s disease on Income over time

**Fig. 3:**
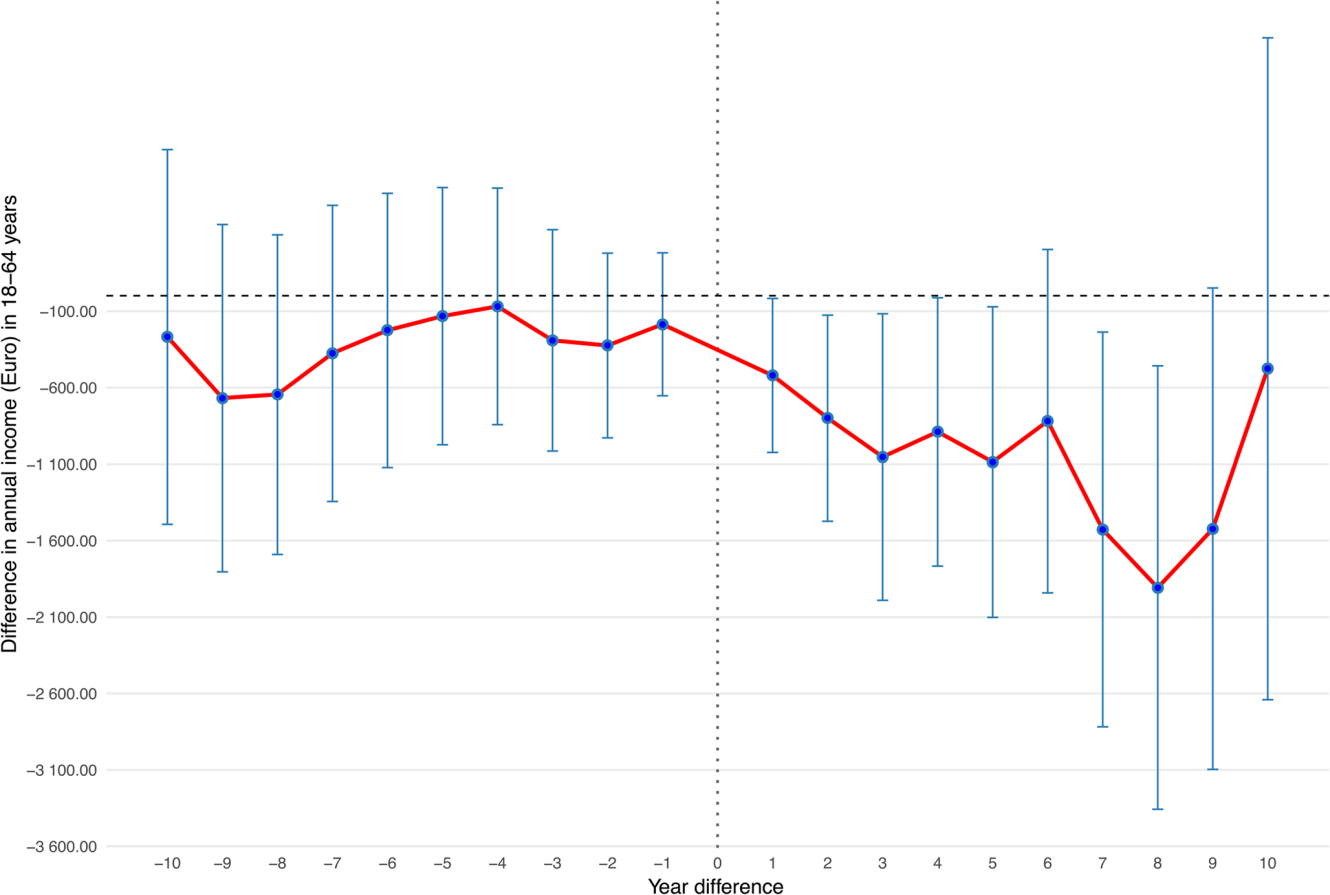
Annual income differences between patients with Ménière’s disease and matched controls aged 18–64, relative to the index year, Denmark 2002–2016

Figure 3 shows the yearly income difference in EUR between people aged 18–64 with Ménière’s disease and controls, based on the interaction term from a linear model.

Before diagnosis, income differences between the two groups were minimal and not statistically significant, with confidence intervals overlapping zero throughout the pre-diagnosis period. However, from the index year, a downward trend appeared. From year 2 onwards, the population with Ménière’s disease consistently earned less on average than their matched counterparts, with the income gap gradually widening over time. By years 7–10, the estimated mean difference in annual income ranged from EUR 1,522 (*p* = 0.058) to EUR 1,907 (*p* = 0.01) lower among those with Ménière’s disease.

For the subpopulation with a CCI of 0, the same trend was observed, with a peak at years 7–10, where values ranged from EUR 1,498 (*p* = 0.04) to EUR 2,008 (*p* = 0.01) lower among those with Ménière’s disease.

Additional stratified plots by age and education on income are available in the supplementary material.

### Use of social benefits before and after diagnosis

**Fig. 4:**
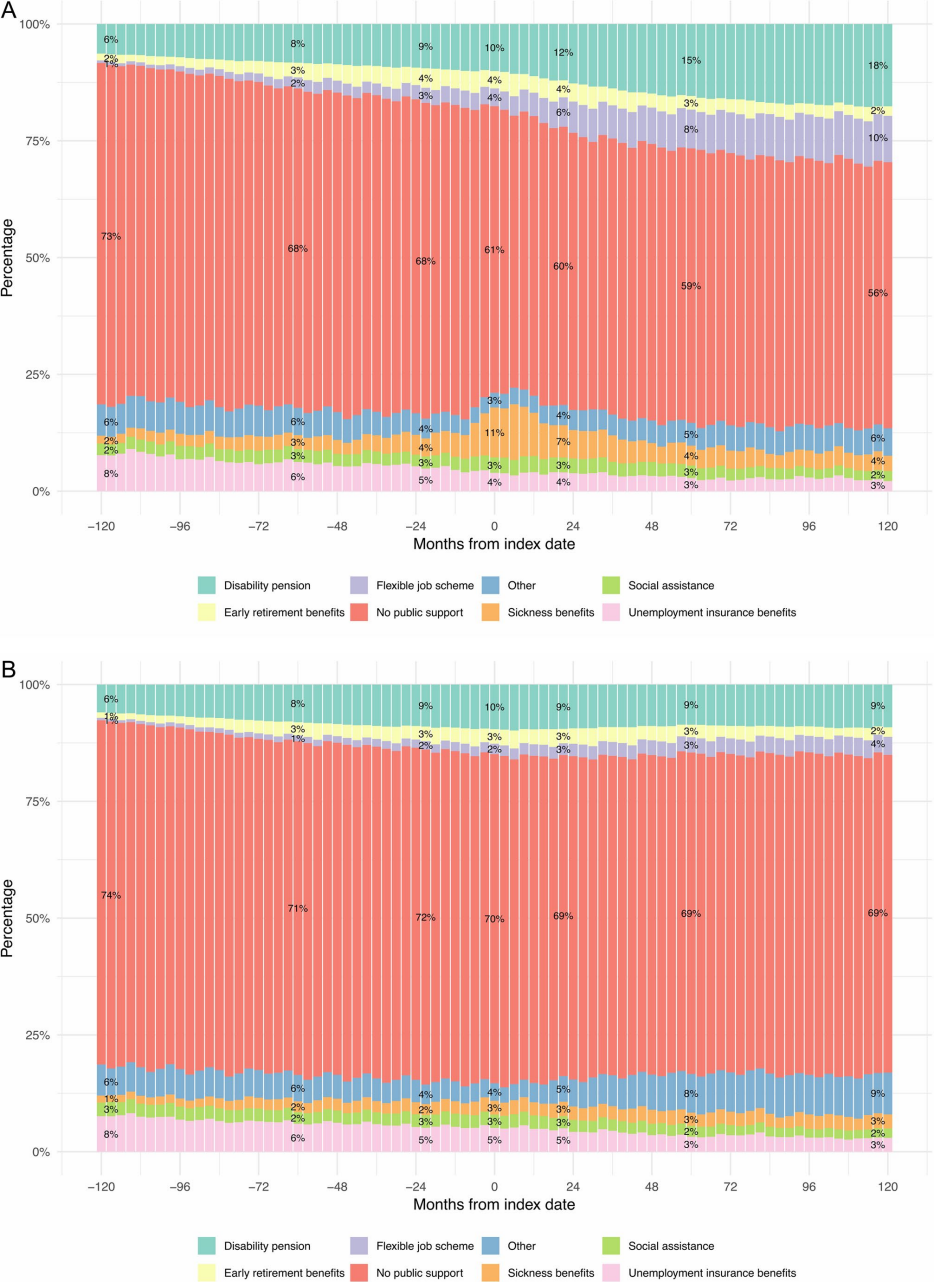
Monthly trends in employment and welfare receipt among individuals with Ménière’s disease (**a**) and matched controls (**b**) aged 18–64, Denmark 2002–2016

Figure 4 illustrates the distribution of public support types over months among individuals aged 18–64 within the two groups. For the controls, the distribution remained relatively stable over time. Around 69–74% of controls consistently did not receive any public support, and the proportion receiving disability pensions remained low, ranging from 6% at −120 months to 9% at + 120 months.

For patients with Ménière’s disease, the proportion without public support decreased over time, from 73% at −120 months to 56% at + 120 months. Disability pension increased significantly from 6% at −120 months to 18% post-diagnosis at + 120 months. The flex job scheme^1^ and early retirement also increased after diagnosis, indicating reduced work ability over time. Sickness benefits peaked around the index year (up to 11%).

For patients with Ménière’s disease in the subgroup with a CCI of 0, the proportion on disability pension increased from 5% at −120 months to 16% at + 120 months. For the flex job scheme, the proportion was 1% at −120 months and 10% at + 120 months. Sickness benefits also peaked around the index year at 10%.

Additional stratification by gender and educational level in the use of social benefits is available in the supplementary.

Eight years after the index year, the multinomial logistic regression showed an OR of 2.32 (95% CI: 2.01–2.70, *p* < 0.001) for disability pension among individuals with Ménière’s disease compared to controls. The odds of being employed in a flexible job scheme were similarly increased, with an OR of 3.23 (95% CI: 2.65–3.93, *p* < 0.001). Please see Supplementary 3.

## Discussion

This nationwide Danish matched cohort study investigated whether individuals diagnosed with Ménière’s disease experienced differences in healthcare use, income, and dependence on social benefits after receiving the diagnosis compared to matched controls. The modest pre-diagnostic differences likely reflect the prolonged clinical course of Ménière’s disease, with symptoms and management in private practice often preceding hospital referral by several years, rather than systematic baseline differences between cases and controls.

We found that individuals diagnosed with Ménière’s disease used more hospital services than controls in the years following diagnosis. This was especially apparent at specialist clinics and hospital outpatient registers. The higher overall healthcare costs in the Ménière’s group were mainly due to increased healthcare utilisation, rather than significantly higher costs per contact, which only increased slightly from two years to one year before diagnosis (Fig. 2). This aligns with a US study reporting substantially higher healthcare costs for Ménière’s disease, primarily driven by frequent hospital outpatient visits [15]. However, that study analysed direct healthcare costs over a single year and included both incident and prevalent cases, as well as patients with other episodic vestibular disorders (vestibular migraine and BPPV), making the study population more diverse than ours. In contrast, our study tracks only newly diagnosed, hospital-confirmed cases of Ménière’s disease over a ten-year period and examines long-term economic and welfare outcomes alongside healthcare use. Moreover, the notably higher annual costs reported in the US probably reflect structural differences between the US healthcare system and Denmark’s universal, tax-funded model, which further limits direct comparison [15].

Furthermore, patients with Ménière’s disease experienced a decline in average annual income after diagnosis, as shown in Fig. 3. The first year may partly reflect temporary sickness benefits. However, under Danish legislation, sickness benefits are limited in time, requiring either a return to work or a shift to other forms of income support. Consequently, in later years, many patients unable to work switch to schemes such as flexible job arrangements or disability pensions, which influences the income pattern over time. A British study similarly found that indirect costs, including loss of income and reliance on social benefits, made up the majority of the economic burden of Ménière’s disease (around 88%), whereas direct healthcare costs accounted for only 12% [17].

Patients with Ménière’s disease were also more likely to receive social benefits over time compared to matched controls. This divergence became most pronounced five years after diagnosis and was especially evident for disability pension, sickness benefits, and participation in flexible work schemes. Our findings suggest that Ménière’s disease may significantly impact work capacity and long-term economic wellbeing in our subgroup. The increasing dependence on public support highlights the chronic and disabling nature of the disease for some patients. This is supported by an international study of 625 patients across 13 countries, which also showed a substantial impact on employment following diagnosis: 72% reported reduced workload, 70% experienced lost workdays due to symptoms, 9.2% had to change jobs, and 8.9% were compelled to stop working altogether [29]. In addition, a study found a statistically significant difference in disability pension among patients compared with controls in the years following the Ménière diagnosis, peaking at 8 years post-diagnosis, with an odds ratio of 2.8. Furthermore, the odds ratio of sickness benefits was 2.3 at the year of diagnosis compared to controls [4].

This study benefits from high-quality national register data with long-term follow-up, enabling near-complete tracking of healthcare use, income, and benefit receipt while minimising bias from loss to follow-up. Patients with Ménière’s disease were matched to controls based on key sociodemographic factors, reducing confounding. By incorporating data spanning 10 years before and after diagnosis, the study can observe long-term patterns and transitions in public support. A notable strength of the Danish healthcare system is the requirement to report a main diagnosis for all hospital contacts as a condition for reimbursement. This guarantees a high level of diagnostic accuracy and significantly reduces the risk of underreporting.

## Limitations

Although matching was performed, residual confounding from unmeasured factors such as health behaviours and symptom severity may still exist. Since the Danish National Health Service register does not include ICD codes, we could not identify patients with Ménière’s disease who had not been referred to public hospital care. Consequently, the index date captures the first hospital registration rather than the first clinical diagnosis. Some patients may therefore have had a clinical diagnosis of Ménière’s disease and received treatment in a private ear, nose, and throat practice for several years before hospital referral, which can explain the observed increases in healthcare utilisation observed in the years preceding the index year. While many patients with Ménière’s disease in Denmark are treated in public hospitals, the population in this study therefore likely represents those with more severe symptoms or greater healthcare needs.

Also, the results should be interpreted in light of Denmark’s welfare system, which includes universal healthcare, sickness benefits, and employment programs such as the Flexible Job Scheme and Disability Pension. These features probably reduce some financial and employment challenges associated with chronic illness more than in countries with less comprehensive welfare support. As a result, the timing and magnitude of income loss, healthcare usage, and reliance on social benefits observed in Denmark might not directly translate to countries with different labour markets, social insurance, or healthcare funding structures. Nonetheless, the overall long-term economic and welfare patterns following Ménière’s disease may remain relevant across various settings. Moreover, the absence of symptom-level data in register sources restricts the ability to evaluate disease burden and functional status.

The diagnostic criteria for Ménière’s disease were revised in 2015, while those for vestibular migraine were established in 2012. As a result, the reported incidence of these conditions may have changed over the subsequent years, with some patients initially suspected of having Ménière’s disease actually suffering from vestibular migraine. Additionally, recent years have seen a shift in treatment practices, with more patients with Ménière’s disease now being managed by office-based specialists rather than being referred to hospitals, compared to earlier periods.

Finally, we did not include medication use, medical devices, or paramedical care costs, as these were not available and would have been relevant for calculating total costs.

## Conclusion

In this nationwide matched cohort study focusing on Ménière’s disease, we found that the condition is associated with a significant and sustained increase in healthcare utilisation and a gradual decline in income over time. While higher healthcare costs were mainly driven by increased frequency of medical contacts, the disease was also linked to long-term economic and labour market consequences. Patients with Ménière’s disease were more likely than matched controls to transition to a disability pension, especially five years after diagnosis, indicating a cumulative impact on work capacity. This study underscores the need for a multidisciplinary approach that addresses not only the disease’s somatic but also its psychological aspects.

## Supplementary Information

Below is the link to the electronic supplementary material.

Supplementary File 1: Total healthcare costs and foregone earnings. (PNG 4.36 MB)

High Resolution Image (TIF 88.9 MB)

Supplementary Material 2: Flowchart of patient enrolment

Supplementary File 3 (PNG 701 KB)

High Resolution Image (EPS 103 KB)

Supplementary File 4: Annual healthcare costs stratified by age groups (PNG 604 KB)

High Resolution Image (EPS 56.4 KB)

Supplementary File 4: Annual healthcare costs stratified by age groups (PNG 722 KB)

High Resolution Image (EPS 103 KB)

Supplementary File 5: Annual healthcare costs stratified by gender (PNG 520 KB)

High Resolution Image (EPS 35.2 KB)

Supplementary File 6: Annual income stratified by age groups (PNG 422 KB)

High Resolution Image (EPS 268 KB)

Supplementary File 6: Annual income stratified by age groups (PNG 312 KB)

High Resolution Image (EPS 107 KB)

Supplementary Material 9: Use of social benefits stratified by gender (patients)

Supplementary Material 10: ICD-codes for Charlson comorbidity index

Supplementary Material 11: Use of social benefits stratified by education

Supplementary Material 12

## Data Availability

The data are available from Statistics Denmark but restrictions apply to the availability of these data, which were used under license for the current study, and so are not publicly available.

## Footnotes

1 The flex job scheme is a publicly funded employment initiative supporting individuals with permanent and significantly reduced work capacity to retain or re-enter the labor market under subsidized conditions.
