# Supplementary material for: Long-term economic and welfare consequences of Ménière’s disease: a Danish nationwide matched cohort study, 2002–2016
Source: Eur Arch Otorhinolaryngol. 2026 May 5;283(7):4287–96. doi: 10.1007/s00405-026-10140-z (PMC13388364; doi:10.1007/s00405-026-10140-z)
Supplement: Supplementary file 3 — Supplementary Material 2: Flowchart of patient enrolment [file 405_2026_10140_MOESM2_ESM.docx]

| y.level | term | estimate | std.error | statistic | p.value | conf.low | conf.high |
| --- | --- | --- | --- | --- | --- | --- | --- |
| Disability pension | caseMeniere's disease | 2.323 | 0.075 | 11.278 | 0.000 | 2.007 | 2.690 |
| Early retirement benefits | caseMeniere's disease | 1.472 | 0.181 | 2.142 | 0.032 | 1.033 | 2.098 |
| Flexible job scheme | caseMeniere's disease | 3.226 | 0.101 | 11.619 | 0.000 | 2.647 | 3.930 |
| Other | caseMeniere's disease | 0.773 | 0.107 | -2.419 | 0.016 | 0.627 | 0.952 |
| Sickness benefits | caseMeniere's disease | 1.462 | 0.134 | 2.830 | 0.005 | 1.124 | 1.903 |
| Social assistance | caseMeniere's disease | 1.303 | 0.177 | 1.496 | 0.135 | 0.921 | 1.843 |
| Unemployment insurance benefits | caseMeniere's disease | 1.162 | 0.148 | 1.020 | 0.308 | 0.870 | 1.552 |
