# Supplementary figures and images for: Long-term economic and welfare consequences of Ménière’s disease: a Danish nationwide matched cohort study, 2002–2016
Source: Eur Arch Otorhinolaryngol. 2026 May 5;283(7):4287–96. doi: 10.1007/s00405-026-10140-z (PMC13388364; doi:10.1007/s00405-026-10140-z)

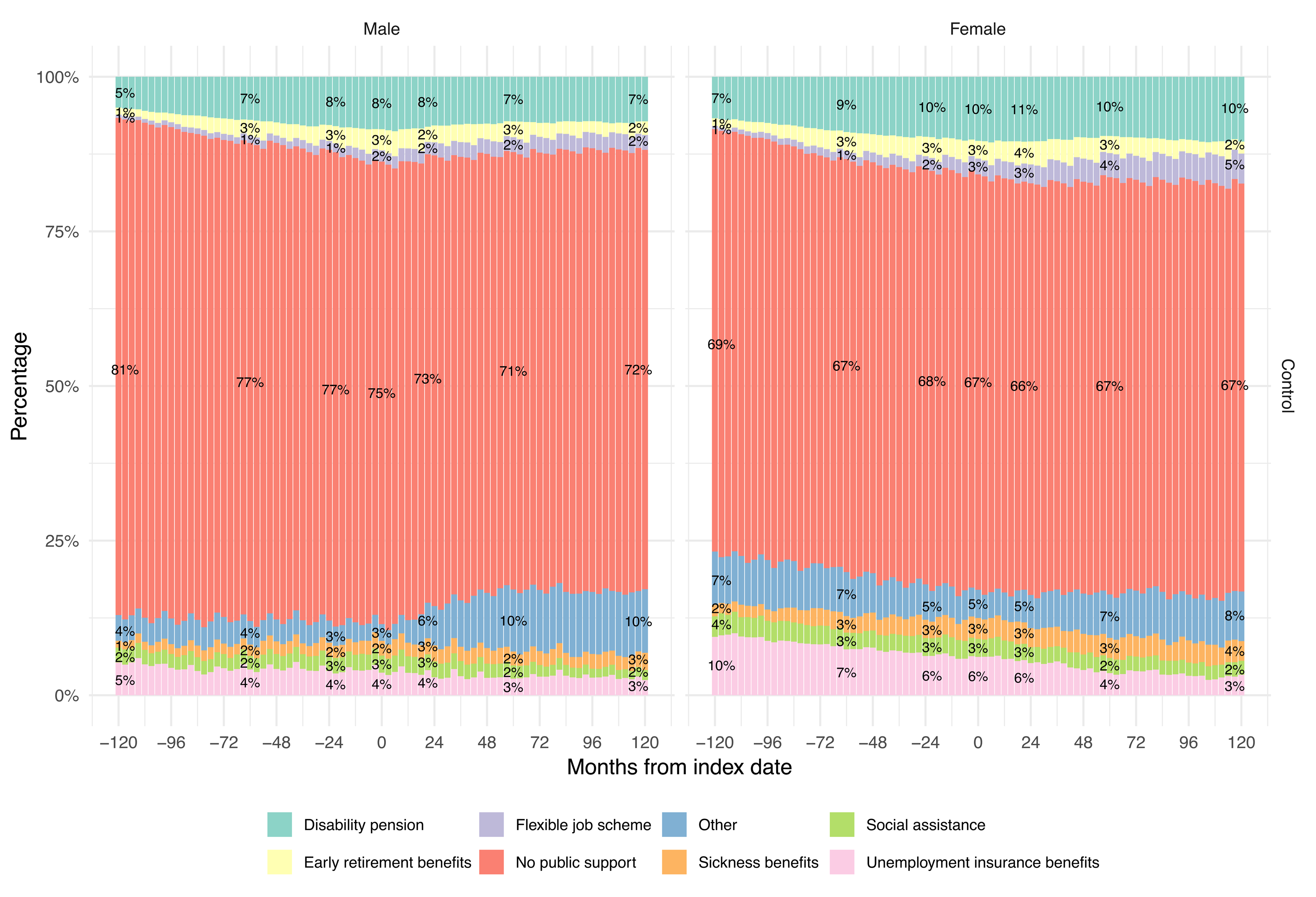

Supplement: Supplementary file 4 — (PNG 701 KB) [file 405_2026_10140_Fig6_ESM.png]

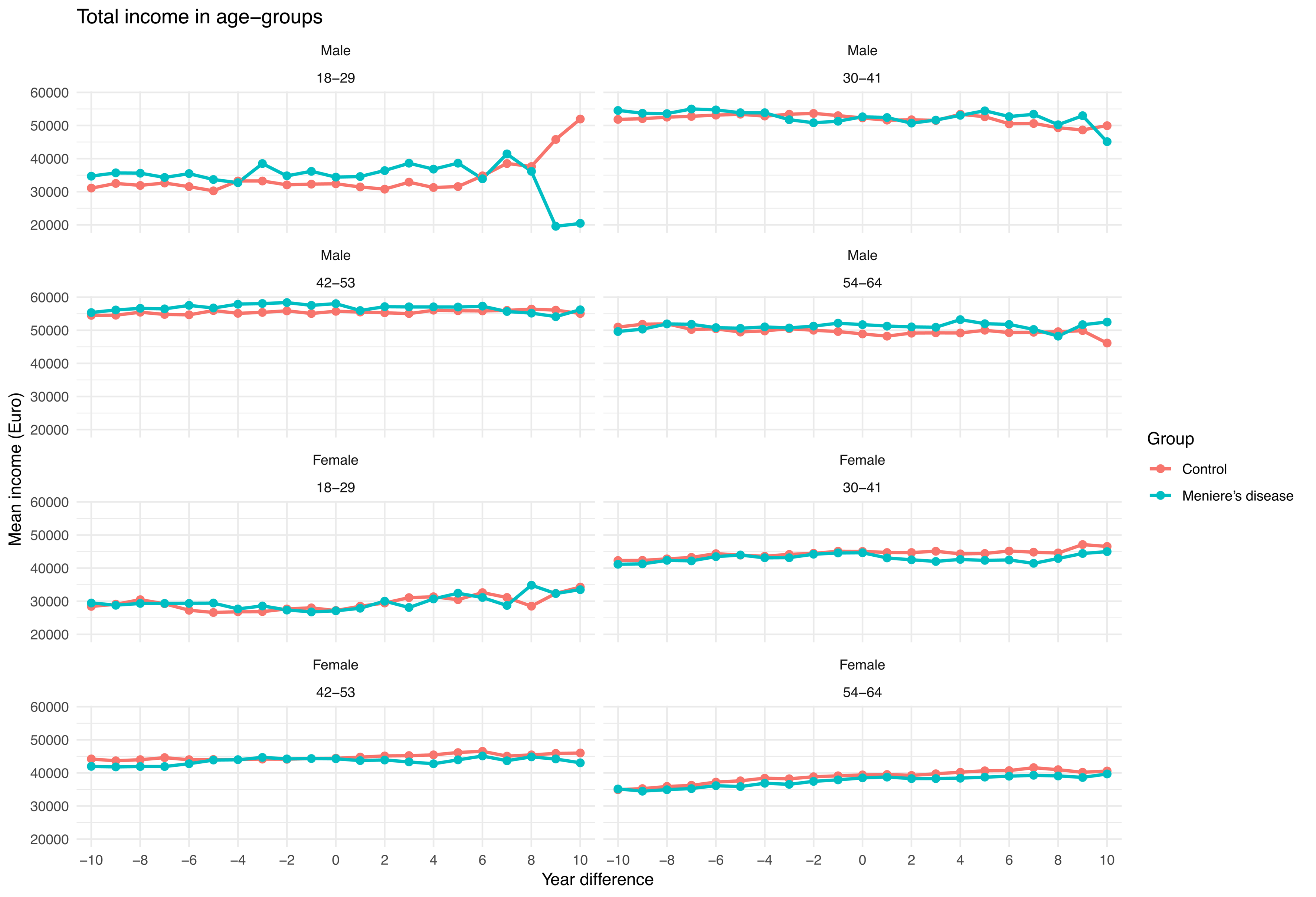

Supplement: Supplementary file 6 — (PNG 604 KB) [file 405_2026_10140_Fig7_ESM.png]

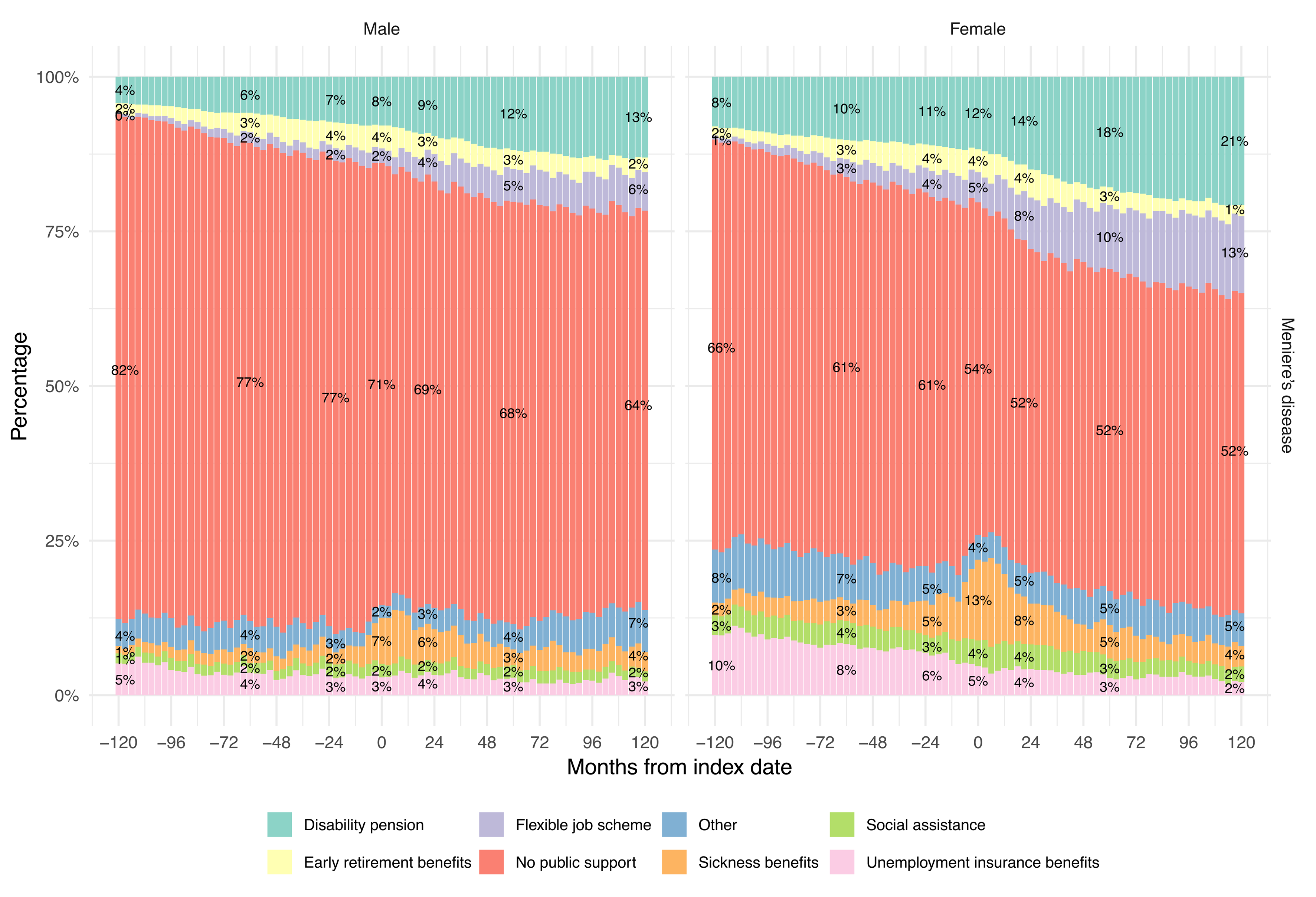

Supplement: Supplementary file 8 — (PNG 722 KB) [file 405_2026_10140_Fig8_ESM.png]

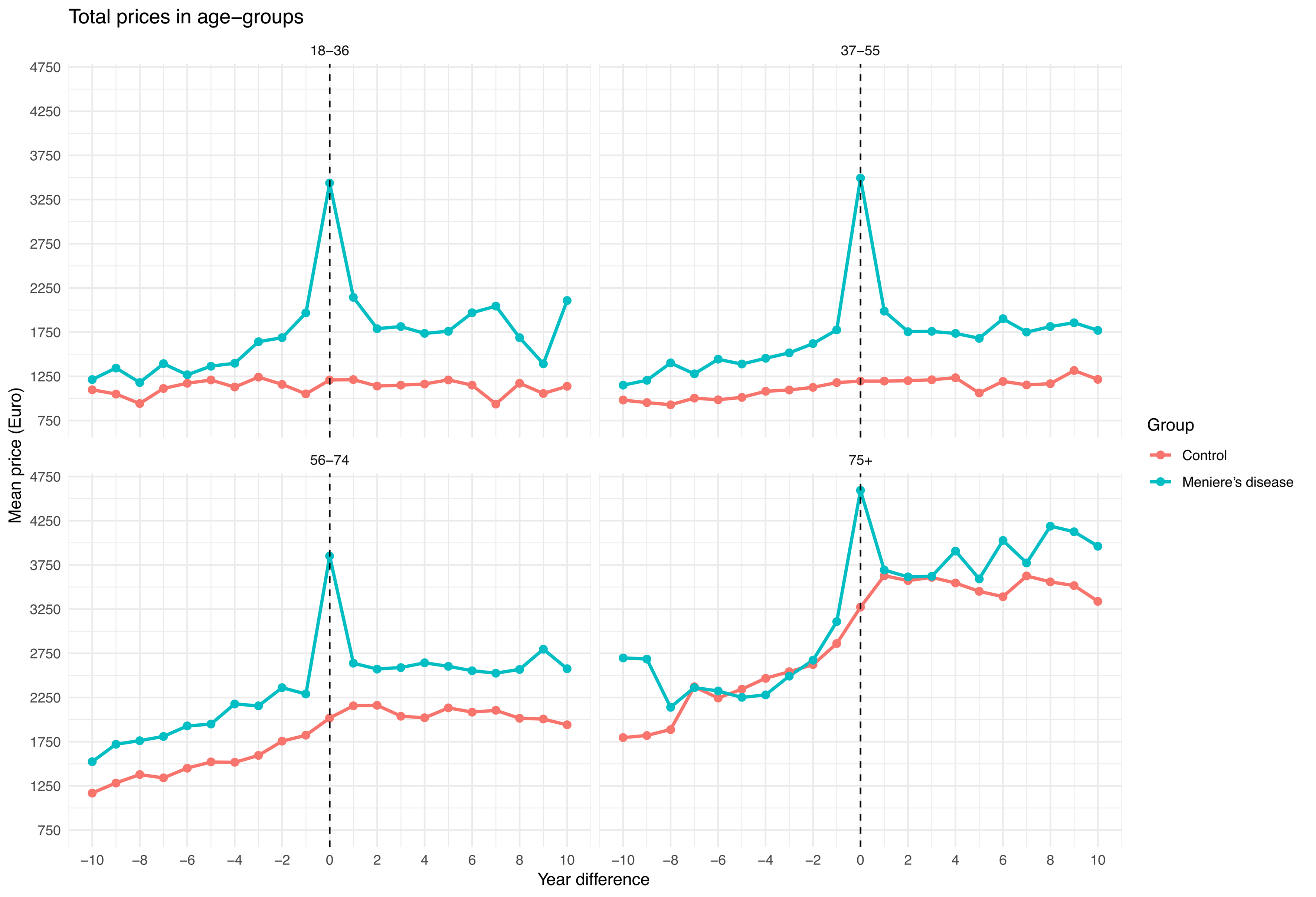

Supplement: Supplementary file 10 — (PNG 520 KB) [file 405_2026_10140_Fig9_ESM.png]

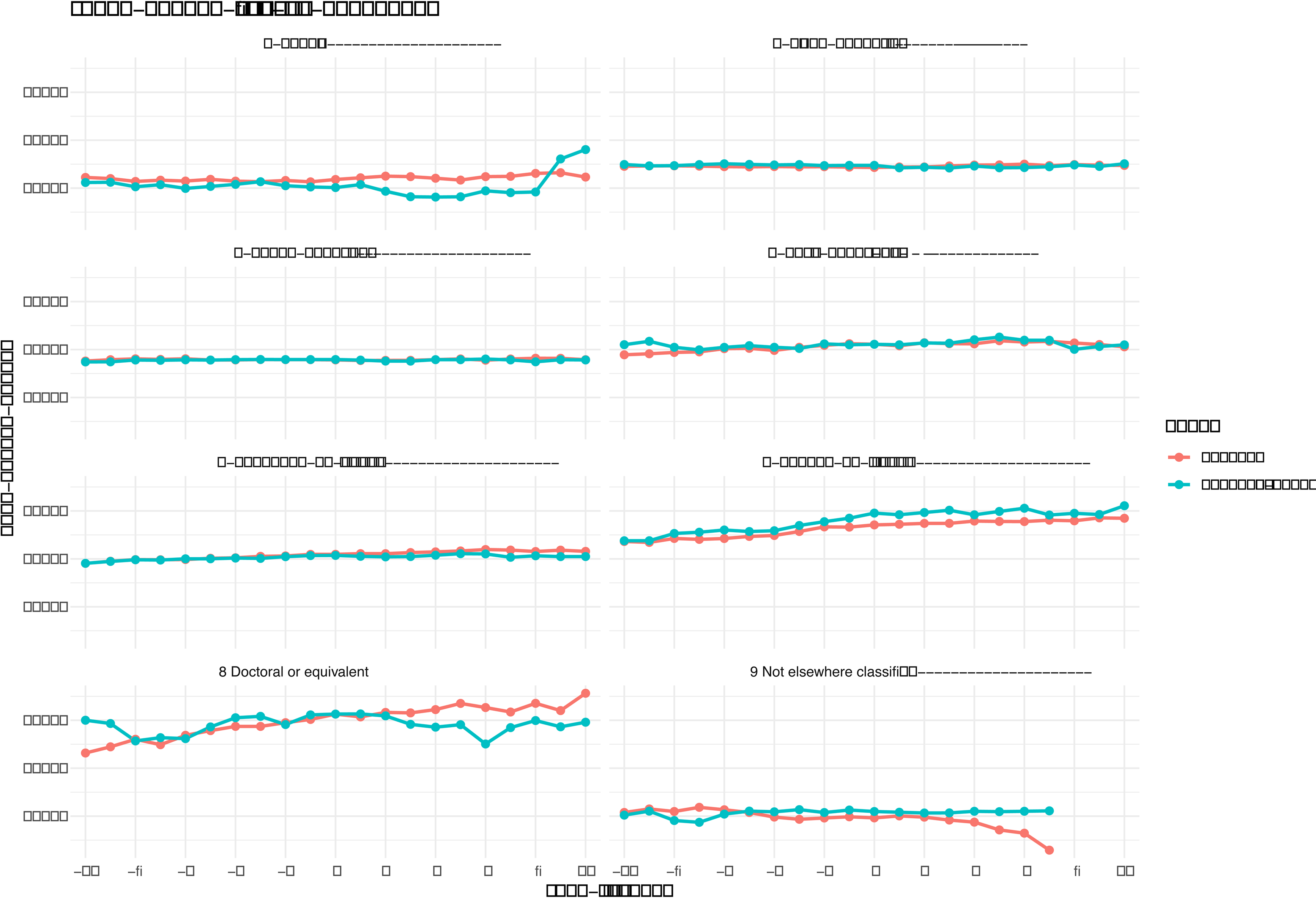

Supplement: Supplementary file 12 — (PNG 422 KB) [file 405_2026_10140_Fig10_ESM.png]

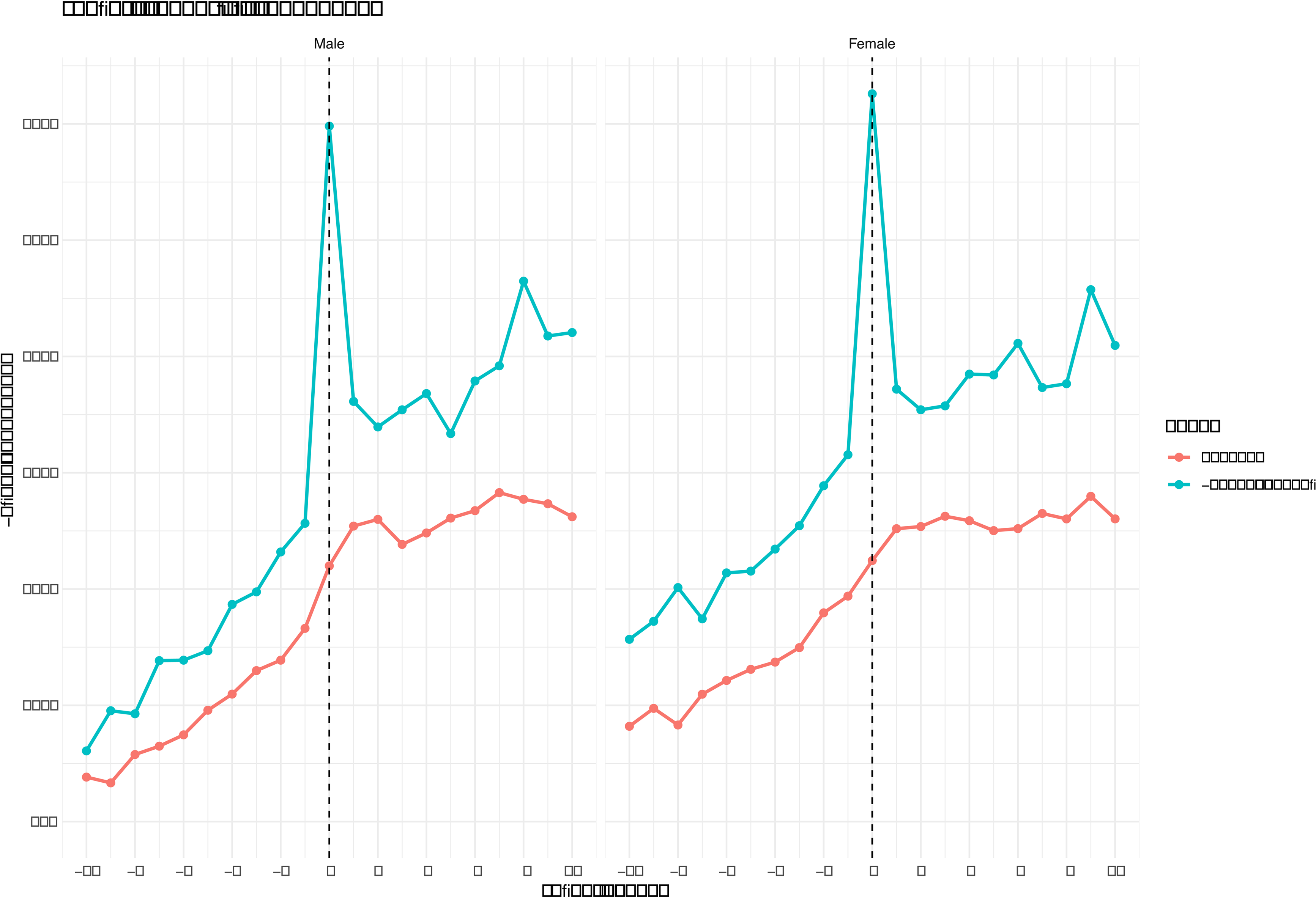

Supplement: Supplementary file 14 — (PNG 312 KB) [file 405_2026_10140_Fig11_ESM.png]

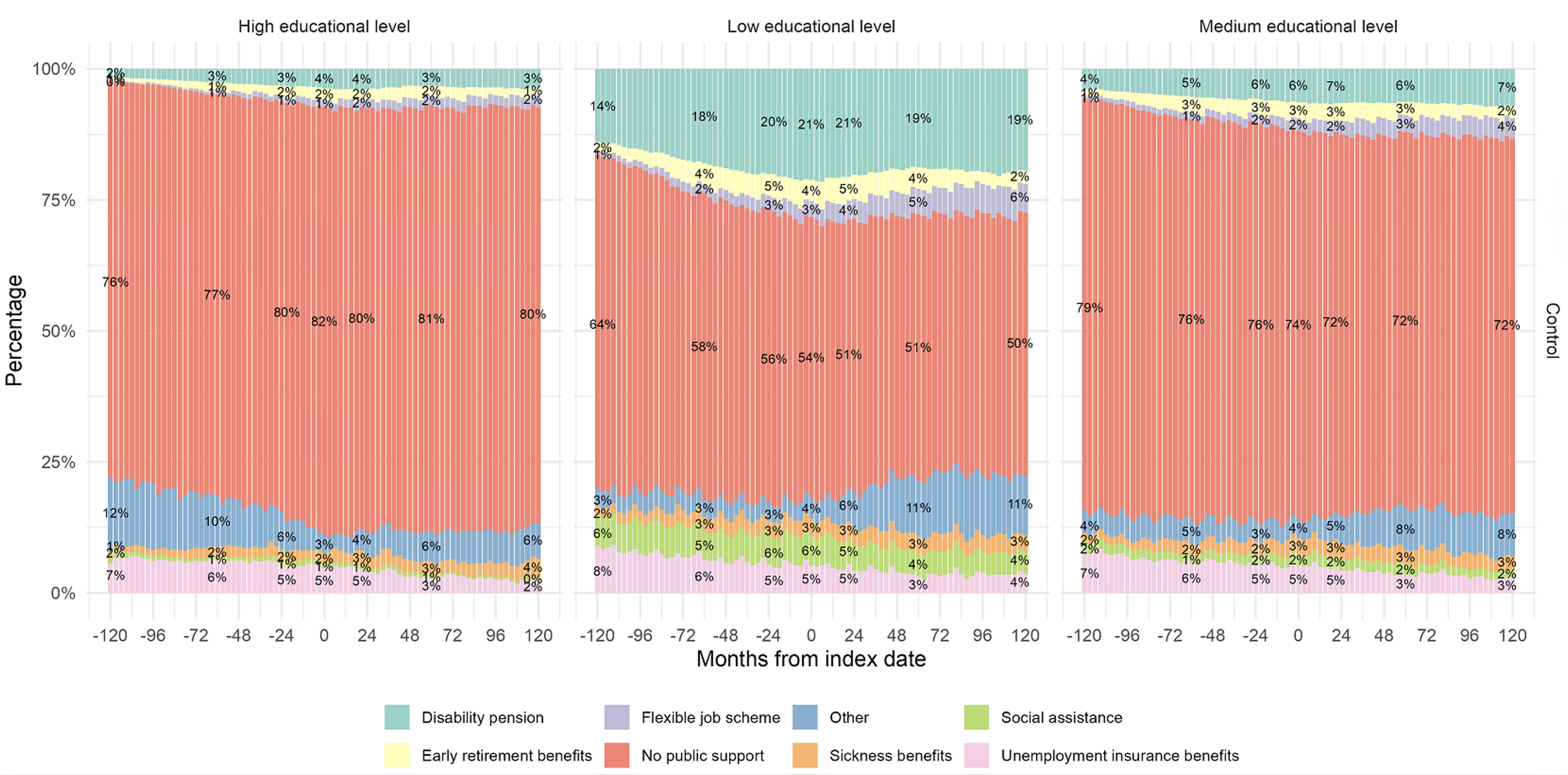

Supplement: Supplementary file 18 — Supplementary Material 11: Use of social benefits stratified by education [file 405_2026_10140_MOESM11_ESM.png]

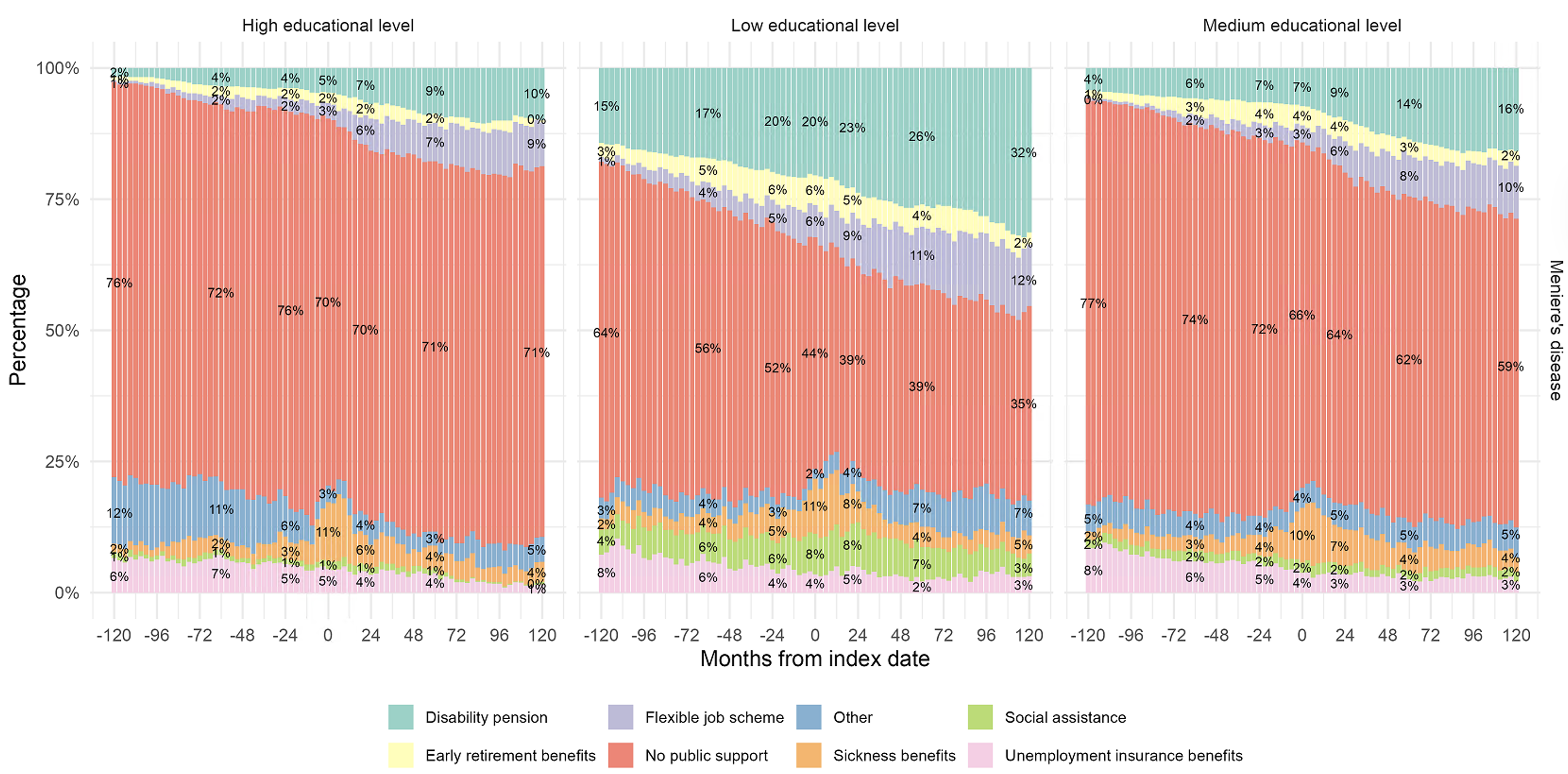

Supplement: Supplementary file 19 — Supplementary Material 12 [file 405_2026_10140_MOESM12_ESM.png]
