# Supplementary material for: Long-term economic and welfare consequences of Ménière’s disease: a Danish nationwide matched cohort study, 2002–2016
Source: Eur Arch Otorhinolaryngol. 2026 May 5;283(7):4287–96. doi: 10.1007/s00405-026-10140-z (PMC13388364; doi:10.1007/s00405-026-10140-z)
Supplement: Supplementary file 16 — Supplementary Material 9: Use of social benefits stratified by gender (patients) [file 405_2026_10140_MOESM9_ESM.docx]

# Supplementary Table. ICD-codes used to define CCI-conditions

| **Charlson condition** | **ICD-8 codes** | **ICD-10 codes** |
| --- | --- | --- |
| Myocardial infarction | 410 | DI21, DI22 |
| Heart failure | 42709, 42710, 42711, 42719, 42899, 78249 | DI099, DI110, DI130, DI132, DI255, DI425, DI426, DI427, DI429, DI428A, DP290, DI43, DI50, DE105, DE115, DE125, DE135, DE145 |
| Peripheral vascular disease | 440–445 | DI70, DI71, DI72, DI731, DI738, DI739, DI77, DI790, DI792, DK551, DK558, DK559, DZ958, DZ959 |
| Cerebrovascular disease | 430–438 | DI60–DI69, DG45, DG46, DH340 |
| Dementia | 290 | DF00, DF01, DF02, DF03, DG30, DF051, DG311 |
| Chronic pulmonary disease | 490–493, 515–518 | DJ40–DJ47, DJ60–DJ67, DJ684, DI278, DI279, DJ84, DJ701, DJ703, DJ920, DJ953, DJ961, DJ982, DJ983 |
| Rheumatic disease | 446, 712, 716, 734, 13599 | DM05, DM06, DM08, DM09, DM30–DM36, D86 |
| Peptic ulcer disease | 53091, 53098, 531–534 | DK25–DK28, DK221 |
| Mild liver disease | 571, 57301, 57304 | DB18, DK700–DK709, DK713–DK715, DK717, DK73, DK74, DK760, DK762–DK764, DK769, DZ944 |
| Severe liver disease | 07000, 07002, 07004, 07006, 07008, 45601–45609, 57300 | DB150, DB160, DB162, DB190, DI850, DI859, DI864, DI982, DK704, DK711, DK721, DK729, DK765–DK767 |
| Diabetes without complications | 24900, 24906, 24907, 24909, 25000, 25006, 25007, 25009 | DE100, DE101, DE108, DE109, DE110, DE111, DE119, DE120, DE121, DE129, DE130, DE131, DE139, DE140, DE141, DE149 |
| Diabetes with complications | 24901–24905, 24908, 25001–25005, 25008 | DE102–DE107, DE112–DE118, DE122–DE128, DE132–DE138, DE142–DE148 |
| Hemiplegia or paraplegia | 344 | DG830–DG834, DG81, DG82, DG041, DG114, DG801, DG802, DG839 |
| Renal disease | 403, 404, 580–584, 59009, 59319, 75310–75319, 792 | DN032–DN037, DN052–DN057, DN18, DN19, DI120, DI131, DI132, DN250, DN26, DZ490–DZ492, DZ940, DZ992 |
| Any malignancy (non-metastatic) | 140–194, 27559 | DC0–DC6, DC70–DC76, DC86, DC97 |
| Metastatic solid tumor | 195–199 | DC77, DC78, DC79, DC80 |
| Leukemia | 204–207 | DC91–DC95 |
| Lymphoma | 200–203, 27559 | DC81–DC85, DC88, DC90, DC96 |
| AIDS / HIV | 07983 | DB20–DB24 |
