# Supplementary material for: Long-term economic and welfare consequences of Ménière’s disease: a Danish nationwide matched cohort study, 2002–2016
Source: Eur Arch Otorhinolaryngol. 2026 May 5;283(7):4287–96. doi: 10.1007/s00405-026-10140-z (PMC13388364; doi:10.1007/s00405-026-10140-z)
Supplement: Supplementary file 17 — Supplementary Material 10: ICD-codes for Charlson comorbidity index [file 405_2026_10140_MOESM10_ESM.docx]

Table: Total costs over time

Comparison between groups

| Group | Year difference | Annual income | Health costs | Foregone earnings | Total costs | Cost difference between groups |
| --- | --- | --- | --- | --- | --- | --- |
| Control | 0 | 38,948 | 1,862 |  | 1,862 |  |
| Meniere's disease | 0 | 38,972 | 3,818 | -24 | 3,794 | 1,931 |
| Control | 1 | 37,930 | 2,126 |  | 2,126 |  |
| Meniere's disease | 1 | 38,118 | 2,622 | -188 | 2,434 | 308 |
| Control | 2 | 38,076 | 2,123 |  | 2,123 |  |
| Meniere's disease | 2 | 37,908 | 2,518 | 168 | 2,685 | 562 |
| Control | 3 | 38,052 | 2,102 |  | 2,102 |  |
| Meniere's disease | 3 | 37,550 | 2,559 | 502 | 3,061 | 959 |
| Control | 4 | 38,035 | 2,111 |  | 2,111 |  |
| Meniere's disease | 4 | 37,265 | 2,698 | 770 | 3,468 | 1,357 |
| Control | 5 | 37,957 | 2,101 |  | 2,101 |  |
| Meniere's disease | 5 | 36,977 | 2,599 | 980 | 3,579 | 1,478 |
| Control | 6 | 37,870 | 2,120 |  | 2,120 |  |
| Meniere's disease | 6 | 37,021 | 2,800 | 849 | 3,648 | 1,528 |
| Control | 7 | 37,313 | 2,195 |  | 2,195 |  |
| Meniere's disease | 7 | 36,265 | 2,698 | 1,048 | 3,746 | 1,551 |
| Control | 8 | 37,270 | 2,159 |  | 2,159 |  |
| Meniere's disease | 8 | 35,682 | 2,887 | 1,587 | 4,474 | 2,316 |
| Control | 9 | 36,704 | 2,214 |  | 2,214 |  |
| Meniere's disease | 9 | 35,391 | 3,027 | 1,313 | 4,340 | 2,126 |
| Control | 10 | 36,154 | 2,127 |  | 2,127 |  |
| Meniere's disease | 10 | 35,408 | 2,873 | 746 | 3,619 | 1,492 |
